# 3D Printed Lab-on-a-Chip Platform for Chemical Stimulation and Parallel Analysis of Ion Channel Function

**DOI:** 10.3390/mi10080548

**Published:** 2019-08-19

**Authors:** Daniel Aschenbrenner, Oliver Friedrich, Daniel F. Gilbert

**Affiliations:** Institute of Medical Biotechnology, Friedrich-Alexander-Universität Erlangen-Nürnberg, 91052 Erlangen, Germany

**Keywords:** 3D printing, ABS, microfluidic, zigzag channel, Ca^2+^ imaging, Fluo-4 AM, TRPV1, capsaicin, HEK293 cells

## Abstract

Functional imaging has been a widely established method for the assessment of ion channel function in vitro. Conventional infrastructure used for in vitro functional analysis of ion channels is typically proprietary, non-customizable, expensive, and requires a high level of skill to use and maintain. 3D desktop printing, which is employed in the rapid prototyping field, allows for quick engineering of alternatives to conventional imaging infrastructure that are customizable, low cost, and user friendly. Here, we describe an ultra-low-cost microfluidic lab-on-a-chip (LOC) device manufactured using acrylonitrile butadiene styrene (ABS) for in vitro functional imaging of ion channels that can quickly and easily be reconstructed using three-dimensional (3D) desktop printing. The device is light weight (<5 g), small (20 mm × 49 mm), and extremely low cost (<EUR 1). We simulate fluidics within the printed channels and assess the suitability of the engineered chamber to generate homogeneous mixtures during solution exchange. We demonstrate the usability of the 3D printed microfluidic device in a case study using Fluo-4-loaded human embryonal kidney-derived (HEK293) cells, recombinantly expressing the capsaicin receptor, transient receptor potential vanilloid receptor type 1 (TRPV1), as a model system. In the case study, we confirm its applicability to solution exchange for chemical stimulation and parallel functional time-lapse fluorescence microscopy-based calcium imaging. We assess the suitability of ABS for culturing HEK293 cells inside the microfluidic LOC, based on qualitative analysis of microscopic transmission light images of ABS-exposed HEK293 cells and confirm the previously reported biocompatibility of ABS. To highlight the versatility of the 3D printed microfluidic device, we provide an example for multiplication of the shown concept within a 3D printed multichannel microfluidic LOC to be used, for example, in a higher throughput format for parallelized functional analysis of ion channels. While this work focusses on Ca^2+^ imaging with TRPV1 channels, the device may also be useful for application with other ion channel types and in vitro models.

## 1. Introduction

Ion channels are involved in a variety of key cellular functions and are considered very attractive targets for therapeutic intervention [1,2,3,4]. Functional analysis of ion channels is typically conducted by electrophysiological means, using either planar or conventional patch-clamping devices [5,6,7], or based on fluorometric readouts and ion-selective fluorescence indicators [8,9,10,11,12,13]. Both approaches involve solution exchange during functional analysis, e.g., for agonist or drug application or for washing in a control solution, and thus require liquid handling infrastructure, such as perfusion systems (electrophysiology) or autosampling robotics (fluorometry). Commercially available systems for solution exchange are characterized by high speed and high precision liquid handling. These systems are considered ideal for automatic solution exchange but are typically proprietary, and thus unfavorable for several reasons. First, its use with pre-existing infrastructure, as well as application and maintenance, requires a substantial level of technical and product-specific skills, and thus is resource intense and not straightforward. Secondly, the possibilities for connections with third-party equipment are typically limited by the hard- and software side, for example, because existing in- and output ports are only sparsely documented, computer programs are a closed source, or the command set is not available. Finally, commercially available systems, as well as required consumables, such as tubing, capillaries or syringes, are mostly cost intense and not available to a broad range of laboratories in low-resource settings. Although there are examples of cost-effective devices, for example, polydimethylsiloxane (PDMS)- or alginate-based microfluidic systems [14,15,16,17,18,19] which allow for automated liquid handling, as well as three-dimensional (3D) printed chambers for maintenance and microscopic observation of cultured cells [20,21], a platform for chemical stimulation and parallel analysis of ion channel function that is reproducible within a short time, scalable to higher throughput screening mode and at the same time an ultra-low-cost device, has not yet been reported. The increasing availability of rapid manufacturing technology, including 3D printing, also adopted by so-called fabrication labs and the “maker movement”, a culture of do-it-yourself (DIY) system design [22,23,24,25,26], allows virtually anyone to quickly and easily engineer devices of reduced cost and complexity.

To overcome the aforementioned limitations of commercially available technology for functional analysis of ion channels, we aimed to develop a microfluidic platform based on a DIY approach that is easily reproducible and, at the same time, affordable to a broad range of research labs. To this end, we intended to employ 3D desktop printing and acrylonitrile butadiene styrene (ABS), a biocompatible thermoplastic, for additive manufacturing. We further aimed to evaluate the device with respect to the fluid dynamics within the printed channels and to assess the suitability of the engineered device to the generation of homogeneous mixtures for solution exchange and chemical stimulation of living cells. In order to demonstrate the applicability of our device to functional analysis of ion channels, we intended to conduct a case study with Fluo-4-loaded human embryonal kidney-derived (HEK293) cells, recombinantly expressing TRPV1 (transient receptor potential vanilloid receptor type 1). TRPV1 is a cation channel mediating pain perception in somatosensory cells. It is activated by temperatures >43 °C, by a change of pH below 6.8 and by capsaicin, the main pungent or hot constituent in chili peppers [27,28]. When activated, the channel conducts an inward-directed, depolarizing cation current, mainly carried by calcium. The resulting increase in the intracellular calcium concentration [Ca^2+^]_i_ is visualized using calcium-sensitive fluorescence indicators, such as Fluo-4 AM. Fluo-4 AM is a non-ratiometric, fluorescent indicator for quantifying intracellular [Ca^2+^]_i_ within a large dynamic range, around a Kd_(Ca^2+^)_ of 345 nM [29]. Its fluorescence intensity increases with increasing [Ca^2+^]_i_. To assess the suitability of the materials, we employed platform production with respect to culturing HEK293 cells inside the microfluidic lab-on-a-chip (LOC) and we also intended to evaluate cellular viability based on visual, i.e., qualitative and morphometric, inspection of ABS-exposed HEK293 cells. Finally, to show that the described technology is scalable and customizable, we aimed to provide an example of a 3D printed multichannel microfluidic LOC to be used, for example, in higher throughput format for parallelized functional analysis of ion channels.

## 2. Materials and Methods

### 2.1. 3D Printed Lab-on-a-Chip Platform

The 3D printed lab-on-a-chip platform in its current design is made up of a total of two 3D printed parts in the following two individual designs: (1) the main compartment and (2) an outlet connector for drainage tubing. The components were purpose designed in CAD software (Autodesk Inventor 2013, Autodesk, Inc., San Rafael, CA, USA) and manufactured from black or white acrylonitrile butadiene styrene (ABS, MakerBot Industries, New York, NY, USA) using a 3D printer (MakerBot Replicator, MakerBot Industries, New York, NY, USA). Printing parameters were set to 100 µm layer height and 60 mm/s extruder speed. These settings were chosen after evaluating different layer heights and extruder speeds, respectively, for platform production using the aforementioned printer. Exemplary printing results, using different settings, are included in Figure S1 in Supplementary Materials and indicate best results with respect to printing layer height and extruder speed for the above-mentioned values. To allow the chamber to be used for culturing cells as well as for parallel chemical stimulation and high-content live-cell imaging, the bottom of the 3D printed chamber was equipped with a glass window. To this end, a standard 20 mm coverslip (Menzel Gläser, Braunschweig, Germany) was glued to the 3D printed plastic part using polydimethylsiloxane (PDMS), namely SYLGARD 184 silicone elastomer (Dow Corning, Midland, MI, USA) and was cured according to the manufacturer’s instructions. The glue was carefully applied in a thin layer around the opening of the cell chamber to avoid blocking of the cell chamber and microfluidic channel.

### 2.2. Pharmacological Reagents

Capsaicin was obtained from Sigma-Aldrich (St. Louis, MI, USA) and was prepared as 100 mM stock in DMSO. Stocks were frozen at −20 °C. The solutions for experiments were prepared from these stocks on the day of recording.

### 2.3. Calcium Indicator

Fluo-4 AM was obtained from molecular probes and was prepared as 10 mM stock in DMSO. Fluo-4 AM stocks were frozen at −20 °C. The solutions for the calcium imaging experiments were prepared from these stocks on the day of recording.

### 2.4. Cell Culture

All experiments were performed on recombinant HEK293 cells cultured in Dulbecco’s modified Eagle’s medium (DMEM, Invitrogen, Carlsbad, CA, USA) supplemented with 10% fetal calf serum and penicillin (100 U/mL)/streptomycin (100 mg/mL) (Sigma-Aldrich, MI, USA). Cells were cultured at 37 °C, 5% CO_2_ in a humidified incubator according to standard procedures and were passaged weekly.

### 2.5. Cell Line

HEK293 cells (CRL-1573™) were purchased from the American Type Culture Collection (ATCC, Manassas, VA, USA).

### 2.6. Transient Transfection of HEK293 Cells for Calcium Imaging Experiments

Approximately 48 h prior to imaging experiments, the cells were seeded into 6 cm dishes (TPP) at a concentration of 10^6^ cells per dish. TRPV1 was transfected with a total cDNA quantity of 1 µg per 6 cm dish. Cells were transfected using the calcium phosphate precipitation method. We have previously published a detailed comparison of five transient transfection methods employed routinely for this purpose [30].

### 2.7. PDL Coating of the 3D Printed Lab-on-a-Chip Platform for Functional Imaging Experiments

Prior to cell seeding, to support cellular adherence on the glass cover slips, the cell chamber of the 3D printed lab-on-a-chip platform was coated with poly-D-lysine (PDL, Sigma-Aldrich, MI, USA). To this end, 20 µL of a freshly prepared solution containing 100 mg/mL PDL was pipetted into the cell chamber of the 3D printed LOC and was incubated for three hours at room temperature. Upon incubation, the chamber was washed three times with ultrapure water and was stored at 4 °C until the experiment.

### 2.8. Cell Seeding for Functional Imaging Experiments

The day before imaging experiments, transiently transfected HEK293 cells, previously dislodged from a 6 cm dish (TPP, Trasadingen, Switzerland) using 0.25% trypsin–EDTA solution (Gibco BRL, Waltham, MA, USA) and resuspended into DMEM, were counted using a hemocytometer (LO Laboroptik GmbH, Friedrichsdorf, Germany) and 35 µL containing approximately 3500 cells were seeded into the 3D printed lab-on-a-chip platform. Cells were cultured overnight at 37 °C, 5% CO_2_ in a humidified incubator according to standard procedures.

### 2.9. Staining of Cells with the Fluorescent Indicator Fluo-4 AM

Approximately 2 h prior to commencement of experiments, the culture medium in the cell chamber was entirely removed and the cells were incubated in 20 μL staining solution, i.e., standard control solution supplemented with 1 µM pluronic F-127 (Life Technologies, Carlsbad, CA, USA) and 5 µM Fluo-4 AM (molecular probes) final concentrations for 1 h at 37 °C, 5% CO_2_. The standard control solution contained (in mM) NaCl 140, KCl 5, CaCl_2_ 2, MgCl_2_ 1, HEPES 10, and glucose 10 (pH 7.4, NaOH). Upon incubation, the staining solution was replaced by 20 µL standard control solution.

### 2.10. Functional Imaging Experiments

**Figure 1:**
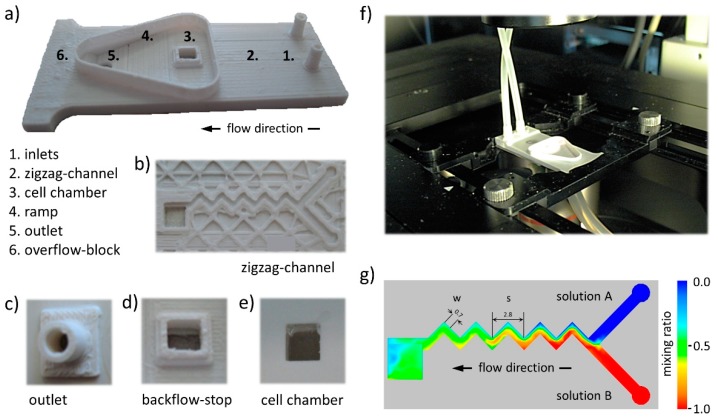
Three-dimensional (3D) printed lab-on-a-chip platform and simulation of fluid dynamics. (**a**) 3D printed lab-on-a-chip platform with inlets (1), zigzag-shaped microfluidics channel (2), cell chamber (3), ramp (4), overflow block (5), and outlet (6). (**b**) View onto the microfluidic zigzag-shaped channel of an incompletely printed chip. (**c**) Outlet for connection with drainage tubing. (**d**) Top view of the cell chamber, surrounded by a backflow stop to support an outward-directed, unidirectional flow of perfused solution. (**e**) Bottom view of the cell culture chamber through a glass window, mounted with a biocompatible silicone elastomer. (**f**) 3D printed lab-on-a-chip platform with connected tubing for functional imaging using a high-content microscope. (**g**) Dimensions of the zigzag-shaped microchannel for generation of homogeneous mixtures, integrating a “Y” junction, with w, the width of the zigzag channel and s, the linear length of the periodic step. False-color representation of simulated fluid dynamics within the platform for two different solutions, ”solution A” (blue) and ”solution B” (red). The green color inside the cell chamber indicates a mixing ration of approximately 0.5, and thus demonstrates homogeneous distribution of efficiently mixed solutions.

Upon termination of cell staining, the 3D printed lab-on-a-chip platform was placed on the motorized stage of a high-content imaging system (Nikon Eclipse Ti, Nikon, Tokyo, Japan) and was imaged with a 10x objective (CFI Plan Fluor DL 10X Phase, NA 0.30, Nikon, Tokyo, Japan). Illumination from a xenon lamp (Lambda LS, Sutter Instruments, Novato, CA, USA), passing through a filter block (C-FL Epi-FL FITC, EX 465-495, DM 505, BA 515-555, Olympus, Tokio, Japan) was used to excite and detect the Fluo-4 fluorescence signal. Fluorescence was imaged with a sCMOS camera (NEO, Andor, Belfast, Ireland) and digitized to a disk on a personal computer (Dell Precision T3500, Dell, Round Rock, TA, USA) running Windows 7 operating System (Microsoft Corporation, Redmond, WA, USA). The primary resolution of the camera was 2560 × 2160 pixel, although images were binned (2 × 2), resulting in a resolution of 1280 × 1080 pixel. The CCD image acquisition rate was 1 Hz. The experimental protocol involved imaging the cells for ten minutes, capturing the fluorescence intensity in the control situation as well as the test situation upon receptor activation, respectively. For TRPV1 receptor stimulation, the standard control solution was supplemented with 1 µM final capsaicin concentration. Capsaicin was diluted from the stock solution at the day of the experiment. Cells were perfused at a rate of 100 µL/min using syringe pumps (Perfusor Compact, B. Braun Melsungen, Melsungen, Germany). The experimental setup is represented in Figure 1f. Imaging experiments were conducted at room temperature.

### 2.11. Single Cell-Based Quantitative Image Analysis

Registered images of fluorescent cells were segmented and quantitatively analyzed using a modified version of DetecTiff^©^ software [31]. The fluorescence signal of identified cells was measured as the mean of all pixel values within the area of a cell.

### 2.12. Data Analysis and Visualization

Plate reader data were annotated in Microsoft Excel and analyzed using Origin 7G (OriginLab Corporation, Northampton, MA, USA).

### 2.13. Simulation of Fluid Dynamics

Fluid dynamics within the microfluidic zigzag channel of our platform were calculated using Autodesk Simulation CFD 2013 (Autodesk Inc., San Raphael, CA, USA). The simulation parameters used were as follows: Perfusion rate, 100 µL/min; density of used solutions, 10 kg/m^3^; diffusion coefficient of capsaicin in aqueous solution, 2 × 10^−6^ cm^2^/s [32].

## 3. Results

### 3.1. Ultra-Low-Cost 3D Printed Microfluidic Lab-on-a-Chip

We have developed an ultra-low-cost microfluidic lab-on-a-chip (LOC) device manufactured using acrylonitrile butadiene styrene (ABS) for in vitro functional imaging of ion channels that can quickly and easily be reconstructed using 3D desktop printing. The platform is shown in Figure 1a. It is made up of the following three different parts: (i) a 3D printed microfluidics chamber, (ii) a 3D printed waste outlet, and (iii) a standard 20 mm cover slip-based glass bottom window for culturing cells and microscopic evaluation. The device allows delivery of two individual solutions via separate inlets (see 1 in Figure 1a). The solutions are mixed in a zigzag-shaped microfluidics channel (see 2 in Figure 1a,b) and lead into a cell culture chamber (see 3 in Figure 1a). Perfused solution leaves the chamber on the top side through a backflow stop (see Figure 1d), preventing bidirectional flow of liquids. Disposal of perfused solution is supported via a ramp (see 4 in Figure 1a) and a waste outlet (see 5 in Figure 1a,c). Fluid leakage from the system is prevented via an overflow block (see 6 in Figure 1a). Microscopic observation is possible through a glass window (see Figure 1e), mounted with a biocompatible silicone elastomer. The complete procedure of microchamber fabrication, including 3D printing, assembly as well as coating to support cellular adhesion to the glass bottom window of the cell culture chamber takes approximately 5 h. The device is light weight (<5 g), small (20 mm × 49 mm) and extremely low cost (<EUR 1). Figure 1f shows an image of the platform installed on an inverted high-content imaging microscope.

### 3.2. Microfluidic Zigzag Channel for Generation of Homogeneous Mixtures

For generation of homogeneous mixtures, we decided to employ a passive, microfluidic zigzag channel-based concept for our platform, because, first, it allows quick mixing without additional components [33,34] and, second, it is suitable for rapid manufacturing using desktop 3D printing. The geometry of the zigzag-shaped microfluidic channel is shown in Figure 1a2 and Figure 1b and schematically represented in Figure 1g. The system features two perpendicular and 1000 µm wide feeders that end with a zigzag channel, integrating 90° angles of 700 µm width (w) and 500 µm height. The linear length of one zigzag pattern, i.e., one periodic step (s, 2800 µm), was chosen to result in a geometry ratio (s/w) of four for the constructed zigzag channel, as this ratio has been reported to yield high mixing efficiencies in microfluidic systems [35]. The overall linear length of the zigzag channel is 14 mm. In order to computationally prove the mixing efficiency of the microfluidic zigzag channel, we simulated fluid dynamics within the platform (see Methods for details). Figure 1g shows a false-color representation of the simulated fluid dynamics within the platform for two individual solutions, ”solution A” (blue) and ”solution B” (red), indicating a mixing ratio of approximately 0.5 inside the cell chamber. These data demonstrate that the device is suitable for generation of homogeneous mixtures, allowing for chemical stimulation of cells, i.e., activation of ion channels during functional imaging.

### 3.3. Chemical Stimulation and Parallel Microscopic Analysis of Ion Channel Function

**Figure 2:**
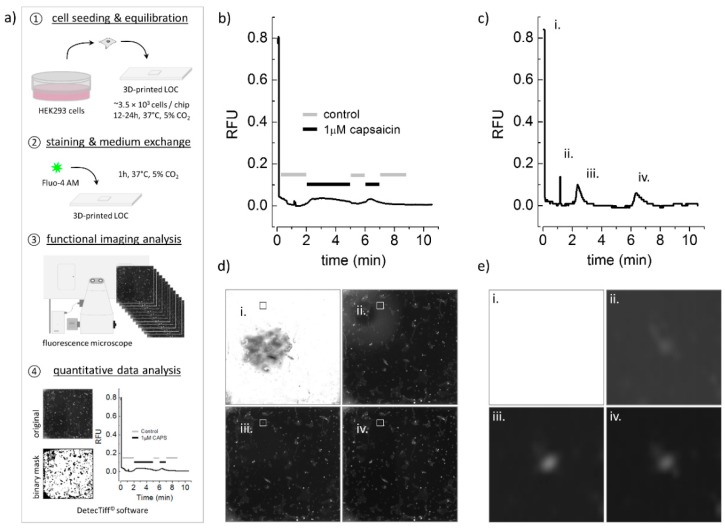
Experimental workflow, as well as chemical stimulation, of transient receptor potential vanilloid receptor type 1 (TRPV1) expressing human embryonal kidney-derived (HEK) cells and parallel fluorescence imaging using the 3D printed lab-on-a-chip platform. (**a**) Experimental workflow for functional ion channel analysis using the 3D printed lab-on-a-chip platform. Details see text. (**b**) Time-course of the averaged and normalized fluorescence signal (RFU, relative fluorescence unit) measured from images taken from fluorescent recombinant cells. (**c**) Time course of the normalized fluorescence signal measured from a single cell, see white frames in (**d**) and images in (**e**). The letters indicate different time points at experiment initiation (i), during perfusion with control solution (ii) for removal of air bubbles from the tubing and microchannels, prior to chemical stimulation, as well as during chemical stimulation with the TRPV1-agonist capsaicin (iii, iv). (**d**) and (**e**) Micrographs of a Fluo-4-loaded HEK cells and a single cell, respectively, taken at different time points during functional analysis as described in (**b**). These data clearly demonstrate that the 3D printed lab-on-a-chip platform is suitable for chemical stimulation and parallel analysis of ion channel function.

To prove that our platform is applicable for chemical stimulation and parallel microscopic analysis of ion channel function, we transiently transfected HEK293 cells with the capsaicin receptor, TRPV1, and used these cells for experimentation with our platform, as detailed in the Methods section and indicated in the workflow in Figure 2a. We used HEK293 (human embryonic kidney-derived) cells because this cell line is a commonly employed in vitro model for addressing a vast variety of biological questions worldwide. As a preparatory step prior to functional imaging, TRPV1-expressing HEK293 cells were pipetted into the cell chamber of the microfluidic platform at a defined number in a standard cell culture medium and were incubated at standard conditions over night (see step 1 in Figure 2a). Upon incubation, the medium was replaced by 20 μL staining solution, i.e., standard control solution, supplemented with pluronic F-127 and Fluo-4 AM and the cells were incubated for 1 h at 37 °C, 5% CO_2_ (see step 2 in Figure 2a). Upon staining, the prepared platform was mounted onto the motorized stage of an automated Nikon Eclipse Ti microscope (see Figure 1f) and the cells were alternatingly perfused with control and test solution, containing 0 and 1 µM capsaicin, respectively. At the same time, they were observed with a fluorescence microscope to measure activation-dependent change in the cellular fluorescence signal (see step 3 in Figure 2a). Acquired images were, subsequently, analyzed using a modified version of DetecTiff^©^ software [29] (see step 4 in Figure 2a). Figure 2 shows time courses of the normalized fluorescence signal, measured from the overall cell population (Figure 2b) and from a single cell (Figure 2c) within the cell chamber. The fluorescence signal increases during perfusion with capsaicin-containing solution, indicating capsaicin-dependent activation of recombinantly expressed TRPV1 channels and a resulting transient rise in [Ca^2+^]_i_. The fluorescence micrographs in Figure 2d,e represent different time points at experiment initiation (i) and during perfusion with a control solution (ii) for removal of air bubbles from the tubing and microchannels, prior to chemical stimulation as well as during chemical stimulation with the TRPV1-agonist capsaicin (iii, iv), taken from the overall cell population (Figure 2d) and from a single cell (Figure 2c). These data clearly prove that the 3D printed lab-on-a-chip platform is suitable for chemical stimulation and parallel fluorescence-based analysis of ion channel function.

### 3.4. Morphology-Based Viability Analysis of HEK293 Cells Cultured Inside the Microfluidic LOC

**Figure 3:**
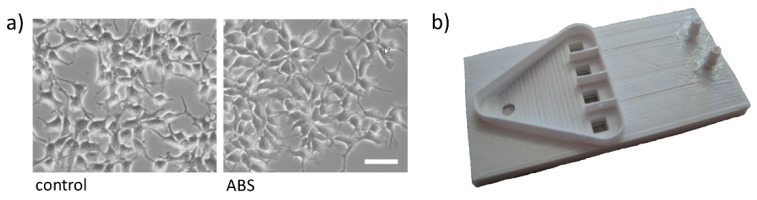
Morphology-based viability analysis of HEK293 cells cultured inside the 3D printed lab-on-a-chip (LOC) and example of multichannel microfluidics platform. (**a**) Bright field images of HEK293 cells cultured in a standard 4 cm culture dish (left, control) and in our microfluidic platform (right, ABS), indicating unaltered morphology, i.e., viability of cells. Scale bar: 50 µm. (**b**) 3D printed demonstrator of a modified microfluidic LOC hosting four instead of a single zigzag channel, as well as cell culture chambers for simultaneous analysis of multiple cell populations within the same experiment.

Despite the fact that all the materials used for the production of the platform, including ABS for the 3D printed parts, glass for the transparent bottom, and PDMS for mounting the glass bottom onto the ABS chip, have been reported to be nontoxic, cytocompatible, and are even partly being used in vivo [36,37,38,39], we aimed to assess potential adverse effects on cellular fitness of these materials by applying the most commonly employed quality control procedure and presumably the gold standard in daily cell culture work, i.e., visual inspection. To this end, we cultured HEK293 cells in 3D printed chambers and standard 4 cm culture dishes for 24 h and, subsequently, imaged the cells using a high-content imaging system. Figure 3a shows representative images of HEK293 cells, indicating unaltered morphology, i.e., viability of cells cultured in our microfluidic platform (ABS) as compared with cells maintained in conventional culture ware (control). Although the presented images provide only qualitative information, these data further highlight the suitability of the presented technology for application with in vitro models, i.e., chemical stimulation and parallel microscopic evaluation of cultured cells.

### 3.5. Example for Multiplication of the Platform in a Multichannel Microfluidic LOC

To highlight the versatility of the platform described in this article, we translated the employed concept into a multichannel microfluidic LOC, hosting four parallel channel systems and allowing for simultaneous analysis of multiple cell populations within the same experiment, to be used, for example, for ion channel research in higher throughput. A 3D printed demonstrator of the multichannel microfluidic platform is shown in Figure 3b.

## 4. Discussion

To address the limitations of commercially available technology suitable to chemical stimulation and parallel microscopic observation of cells in vitro, we have designed a 3D printable lab-on-a-chip platform for chemical stimulation and parallel analysis of ion channel function that is advantageous for several reasons. First, with a small footprint (20 mm × 49 mm) and a mass of less than 5 g, including 3D printed parts and a glass window, the device is usable with any type of inverted or upright microscope. Due to the fact that special holders or adaptors are not required and also its small weight, the platform may also be applicable for mobile use. Secondly, with <EUR 1 material costs, the platform is readily applicable to a broad range of laboratories in various research fields. The device can be reused upon disinfection with, for example, 70% ethyl alcohol, potentially further reducing the costs as compared with commercially available reusable or even disposable stimulation chambers. Third, the lab-on-a-chip system was produced using a conventional desktop 3D printer and can easily be rebuilt in so called ”fab labs” (fabrication laboratories). STP and STL files will be provided on request, allowing for custom modification to meet specific and individual requirements, for example, parallelized ion channel research in higher throughput format. Fourth, as the platform is built from biocompatible materials, including ABS, silicone elastomer, as well as glass, it is applicable for maintenance of cultures of living biological specimens. Finally, the technology in its current configuration is straightforward, easy to use, and does not require highly skilled staff for application and maintenance, thus, further highlighting the applicability in a large spectrum of scientific fields.

Despite the aforementioned advantages over conventional infrastructure, our platform also has the potential for improvement. For example, although the device in the current configuration has proven to be suitable for chemical stimulation and parallel live cell high content microscopic imaging, is has not been optimized or characterized in detail with regard to generation of varying reagent mixtures or gradients, for example, for concentration response experimentation or with respect to perfusion with varying rates. However, although application of varying reagent mixtures was not intended in the presented study, we have simulated fluid dynamics as described in the Methods section using different perfusions rates between 100 and 900 µL/min. Figure S1g in the Supplementary Material shows simulation results for different perfusion rates, clearly demonstrating the impact of the flow rate on regent mixtures in the cell chamber. These data indicate that the presented platform is suitable for the generation of graduated chemical concentrations, to be used, for example, in concentration-response experiments with ion channels. Our main intention was to design and apply a microfluidic LOC platform for chemical stimulation and parallel microscopic analysis of ion channel function that can be fabricated using a 3D printer and that is customizable and extendable for a specific use as well as to prove its applicability for fluorescence-based physiological evaluation of ion channels. With the system now at hand, however, this opens a plethora of functional drug screening studies in future applications.

In order for the platform to be applied in basic research, we recommend further evaluation of the cytocompatibility in long-term experiments and to assess cellular fitness as well as the physiological properties of cultured and chemically stimulated cells for several days or even weeks using independent methodological approaches.

It is important to mention that the applicability of the platform described here depends on the specific experimental setup, on the available instrumental infrastructure, including an automated microscope and a syringe pump or gravimetric system for solution delivery, as well as the biological questions to be addressed. The printed platforms shown in Figure 1 and Figure 3 were manufactured using white ABS. However, for the fluorescence-based approaches we strongly recommend the use of black ABS for printing to reduce auto-fluorescence and light scattering, minimizing contamination with background light, and increasing signal-to-noise-ratio in fluorescence images. In addition, it is crucial to state that the printing settings affect the surface roughness and, consequently, the overall printing accuracy. The higher the printing accuracy and the smaller the surface roughness, the smaller the channel width and height that could be achieved using this method. To minimize surface roughness, we strongly recommend printer-specific optimization for platform production. Optimization also strongly reduces the rejection rate, which in our case was approximately 10% post-optimization. We have optimized the roughness of printed surfaces by testing different layer heights and extruder speeds (see Figure S1 in the Supplementary Material) and found that a layer height of 100 µm and an extruder speed of 60 mm/s reveals the best results. In this configuration, channels of 1000 µm height and 500 µm width were manufactured with high reproducibility. Although smaller dimensions could also be manufactured using this approach, the limitations of the employed method with respect to the smallest possible channel dimensions have not been analyzed in detail in the course of this study. Dimensional variations were not evaluated in detail in the course of this study, however, the variation in all three dimensions is likely in between the positioning characteristics of the employed printer (XY, 11 µm and Z, 2.5 µm as indicated by the manufacturer) and the layer height, which in our case was 100 µm. When assuming a variability of approximately 50 µm in X, Y, and Z direction, this value accounts for five to ten percent of the width and height of the zigzag channel, respectively. An important limitation of the presented lab-on-a-chip device arising from the manufacturing approach using 3D printing and thermoplastic is that it is not suitable for mass production.

Although this work focuses on chemical stimulation and microscopy of recombinantly expressed proteins in mammalian cells, the presented technology could also be applied for in vitro culture and stimulation of cells from other organisms as well as tissues, organs, or even whole multicellular organisms.

In summary, the 3D printed microfluidic lab-on-a-chip platform presented in this article has the power to improve conventional technologies in terms of customizability, user friendliness, and cost. In addition, it provides an example of 3D printable low-cost microfluidics technology that is compatible with quick and resource-efficient prototyping in a vast variety of scientific fields. Altogether, this work contributes to advancing the availability and applicability of three-dimensional printable microfluidic devices for use in biomedical research.

## Supplementary Materials

The following are available online at https://www.mdpi.com/2072-666X/10/8/548/s1: Figure S1, Optimization of printing settings for production of 3D printed lab-on-a-chip platform and simulation of fluid dynamics with different perfusion rates.
