# Supplementary material for: 3D Printed Lab-on-a-Chip Platform for Chemical Stimulation and Parallel Analysis of Ion Channel Function
Source: Micromachines (Basel). 2019 Aug 19;10(8):548. doi: 10.3390/mi10080548 (PMC6722671; doi:10.3390/mi10080548)
Supplement: Supplementary file 1 [file micromachines-10-00548-s001.pdf]

micromachines

Supplementary Information

3D-Printed Lab-on-a-Chip Platform for Chemical Stimulation and Parallel Analysis of Ion Channel Function

Daniel Aschenbrenner, Oliver Friedrich and Daniel F. Gilbert

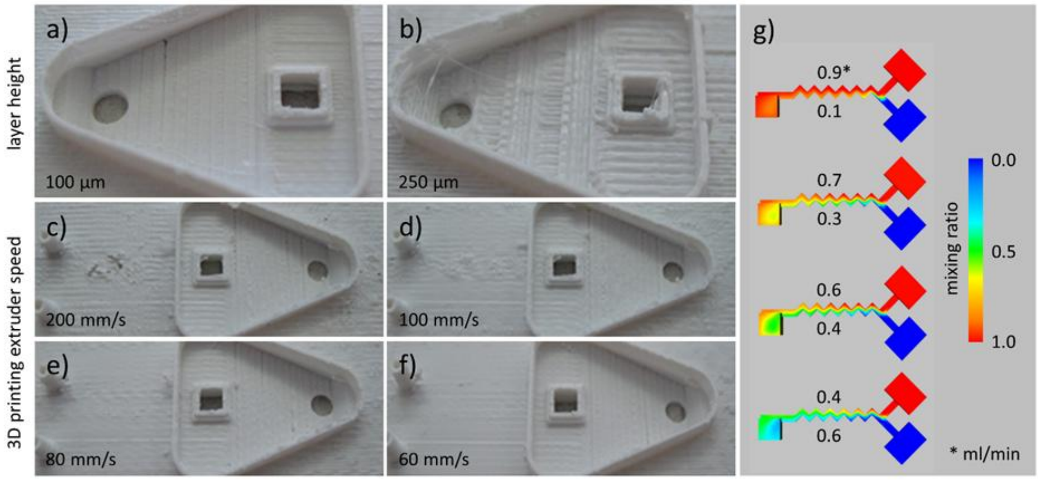

Figure S1. Optimization of printing settings for production of 3D printed lab-on-a-chip platform and simulation of fluid dynamics with different perfusion rates. (**a**,**b**) Images of 3D printed devices using different layer heights. Printing of the lab-chip using a layer height of 100 µm (**a**) revealed better results compared to printing using a layer height of 250 µm (**b**). Platform production was carried out using the smallest possible layer height of 100 µm. (**c**–**f**) Evaluation of the extruder speed for optimized printing of the lab-chip. Fast extruder speeds result in shorter printing times but also reveal larger variation and reduced printing quality. For production of the 3D printed lab-on-a-chip platform 60mm/s extruder speed was used. (**g**) Simulation of fluid dynamics with different perfusion rates, clearly demonstrating the impact of the flow rate on regent mixtures in the cell chamber. These data indicate that the presented platform is suitable for generation of graduated chemical concentrations, to be used e.g., for concentration-response experiments with ion channels.

© 2019 by the authors. Licensee MDPI, Basel, Switzerland. This article is an open access article distributed under the terms and conditions of the Creative Commons Attribution (CC BY) license (http://creativecommons.org/licenses/by/4.0/).
